# Classification of Neurons in the Primate Reticular Formation and Changes after Recovery from Pyramidal Tract Lesion

**DOI:** 10.1523/JNEUROSCI.3371-17.2018

**Published:** 2018-07-04

**Authors:** Boubker Zaaimi, Demetris S. Soteropoulos, Karen M. Fisher, C. Nicholas Riddle, Stuart N. Baker

**Affiliations:** Institute of Neuroscience, Medical School, Newcastle University, Newcastle upon Tyne NE2 4HH, UK

**Keywords:** clustering, lesion, recovery, reticular formation, spikes

## Abstract

The reticular formation is important in primate motor control, both in health and during recovery after brain damage. Little is known about the different neurons present in the reticular nuclei. Here we recorded extracellular spikes from the reticular formation in five healthy female awake behaving monkeys (193 cells), and in two female monkeys 1 year after recovery from a unilateral pyramidal tract lesion (125 cells). Analysis of spike shape and four measures derived from the interspike interval distribution identified four clusters of neurons in control animals. Cluster 1 cells had a slow firing rate. Cluster 2 cells had narrow spikes and irregular firing, which often included high-frequency bursts. Cluster 3 cells were highly rhythmic and fast firing. Cluster 4 cells showed negative spikes. A separate population of 42 cells was antidromically identified as reticulospinal neurons in five anesthetized female monkeys. The distribution of spike width in these cells closely overlaid the distribution for cluster 2, leading us tentatively to suggest that cluster 2 included neurons with reticulospinal projections. In animals after corticospinal lesion, cells could be identified in all four clusters. The firing rate of cells in clusters 1 and 2 was increased in lesioned animals relative to control animals (by 52% and 60%, respectively); cells in cluster 2 were also more regular and more bursting in the lesioned animals. We suggest that changes in both membrane properties and local circuits within the reticular formation occur following lesioning, potentially increasing reticulospinal output to help compensate for lost corticospinal descending drive.

**SIGNIFICANCE STATEMENT** This work is the first to subclassify neurons in the reticular formation, providing insights into the local circuitry of this important but little understood structure. The approach developed can be applied to any extracellular recording from this region, allowing future studies to place their data within our current framework of four neural types. Changes in reticular neurons may be important to subserve functional recovery after damage in human patients, such as after stroke or spinal cord injury.

## Introduction

The reticular formation plays an important role in primate upper limb movements. Reticulospinal connections can activate shoulder and elbow muscles bilaterally (Davidson and Buford, 2004, 2006), as well as forearm and intrinsic hand muscles (Riddle et al., 2009). Reticular activity modulates during independent finger movements as strongly as in the hand representation of primary motor cortex (M1; Soteropoulos et al., 2012). Many cervical spinal interneurons receive convergent input from both the reticulospinal tract (RST) and corticospinal tract (Riddle and Baker, 2010). After a corticospinal lesion, reticulospinal connections to motoneurons innervating the forelimb strengthen (Zaaimi et al., 2012) and may, therefore, be partially responsible for functional recovery (Filli et al., 2014; Baker and Perez, 2017; Owen et al., 2017) and its limitations (Dewald et al., 1995; Miller and Dewald, 2012; Baker et al., 2015; Xu et al., 2017; McPherson et al., 2018; Zaaimi et al., 2018).

Extrinsic connections of the reticular formation have been well studied. Reticulospinal outputs use not only glutamate, but also GABA and glycine (Du Beau et al., 2012; Huma et al., 2014). Reticular nuclei integrate inputs from many cortical (Rossi and Brodal, 1956; Kuypers, 1958) and subcortical (Alstermark et al., 1992; Leiras et al., 2010) sources. These inputs provide the potential to manipulate reticular activity and may provide the opportunity to induce plastic changes in outputs (Foysal et al., 2016), which is of possible use in rehabilitation.

By contrast, much less work has been devoted to understanding the intrinsic circuitry of the reticular formation, and the following statement from Rossi and Brodal (1956) still holds true today: “Knowledge of the minute structure of the reticular formation is still insufficient to permit satisfactory correlations with the many new physiological discoveries.” Classification of the different reticular nuclei is still based on the fraction of large cells present, with little consideration for variations in electrophysiological, morphological, or molecular phenotype. This lacuna in our knowledge is stark when compared with the significant progress made in recent years to subclassify neurons in the cerebral cortex (Ascoli et al., 2008) based on intracellular recordings (Gray and McCormick, 1996; Dégenètais et al., 2002), staining for specific calcium-binding proteins (e.g., parvalbumin; Condé et al., 1994; Gabbott and Bacon, 1996), and patterns of gene expression (Poulin et al., 2016). Knowing what different cell types are present is the first stage toward understanding the computational processes that a neuronal circuit might carry out. We cannot hope to understand the role of the reticular formation in motor control, or how its network changes to aid or hinder recovery after lesion, until we have better knowledge of the different cell classes, their local interconnections, and their specific input/output links with other structures.

Classification of neurons based on extracellular recordings is sometimes possible, most commonly using the width of the action potential. In the cortex, narrow spikes are typically produced by inhibitory interneurons (Mountcastle et al., 1969), and in the rat this can successfully separate excitatory and inhibitory cells (Barthó et al., 2004). However, in primate motor cortex, fast corticospinal cells (which are all excitatory) also have narrow spike widths comparable to inhibitory interneurons, so that absolute distinctions are not always possible (Vigneswaran et al., 2011).

Following a spike, neurons activate conductances that control the afterspike membrane potential trajectory. A delayed depolarization can lead to a further spike in close proximity to the first and a cell with a propensity to burst. This can be detected in extracellular records as a high fraction of short interspike intervals (ISIs; Holt et al., 1996; Bastian and Nguyenkim, 2001). Neurons that do not burst vary in the regularity of their discharge, from clock-like intervals with low variability to highly irregular spiking akin to a Poisson process. Some measures of spiking regularity can remove the impact of slow rate variations seen, for example, as an animal performs a task (Shinomoto et al., 2005; Davies et al., 2006). More sophisticated analysis of ISIs can extract an estimated afterspike distance-to-threshold trajectory (DTT; Matthews, 1996). Typically, this rises monotonically back toward threshold, but some cells have a trajectory peak that can produce regular firing (Wetmore and Baker, 2004; Witham and Baker, 2007).

In this article, we apply these measures to a large dataset of extracellular recordings from the primate reticular formation. We find evidence for four different cell types, one of which may include reticulospinal neurons. These clusters could also be identified in animals that had recovered after a corticospinal tract lesion, but some cell types changed their firing, suggesting that reticular neurons may modify their intrinsic cellular properties as part of functional recovery.

## Materials and Methods

### 

#### Animals

Recordings from awake behaving animals are reported from seven adult female *Macaca mulatta* monkeys; of these, two underwent unilateral lesions of the left pyramidal tract (ages, 4.5, and 4.9 years; weights, 4.5 and 5.7 kg) and five provided control data (age range, 3.5–7.0 years; weight range, 4.3–6.1 kg). Recordings from terminally anesthetized animals are reported from five female *Macaca mulatta* monkeys (age range, 4.4–16.2 years; weight range, 5.6–10.5 kg). All experimental procedures were performed under UK Home Office licenses and were approved by the Newcastle University Animal Welfare and Ethics Review Board.

#### Behavioral tasks

This article combines data recorded for multiple unrelated studies in our laboratory. Several behavioral tasks were used; these data are combined as the relationship of neural activity with behavior was not the primary interest of this report. The tasks are briefly described below.

Monkeys D and R were trained to perform isolated index finger flexion/extension movements. The index finger was placed into a rigid tube, which limited movement to the metacarpo-phalangeal joint, and pivoted about an axis coincident with that joint. Finger displacement was detected by an optical encoder attached to the tube, and torque in an extension direction was applied by a motor activated to simulate a spring. Finger movements were reflected in an on-screen cursor; the animal was required to track a target, producing a hold-ramp-hold pattern of finger flexion or extension before obtaining a food reward. The task has been fully described in our previous publications, which reported data from these animals (Williams et al., 2009, 2010; Soteropoulos et al., 2012).

Monkeys L, G, and P were trained to perform a food retrieval task requiring dexterous right-hand and finger movements. A clear plastic chamber housing a food well was placed in front of the animal and baited by the experimenter with small pieces of fruit. The well was initially kept out of reach by a pneumatically operated clear plastic door. A trial was initiated by the monkey pulling a lever; this caused the door to drop rapidly, allowing the animal to reach into the chamber and retrieve the food. The shape and size of the well, and the soft nature of the reward, necessitated accurate hand shaping followed by fine finger movements under visual and proprioceptive guidance for successful retrieval. Hand entry into the food well was registered by infrared beams, yielding the “pick” event used for the alignment of neural activity. Once the hand left the well, the clear plastic door rose slowly, ending the trial. Monkeys L and G contributed to a previous publication using this task but reporting spinal cord recordings (Riddle and Baker, 2010). Monkey O was trained on this task, but after corticospinal tract lesioning did not regain sufficient dexterity to perform all aspects. This animal instead performed a simpler task, involving grasping a disc placed on a sliding rod. A food reward was positioned on top of the disc. After the disc was grasped, an elbow flexion movement pulled the rod up at an angle of 45° to the horizontal plane, bringing the food to the mouth.

Monkey S was trained to grasp the handle of a robotic manipulandum, which was free to move in a horizontal plane. After the handle was grasped, the robot moved the hand passively in a random trajectory. At the end of this sequence, one of two pots located at the left side of the monkey opened so that the monkey could reach for the food contained inside.

#### Pyramidal tract lesion

Following behavioral training, two monkeys (P and O) underwent a unilateral corticospinal tract lesion, using methods that we have previously described (Zaaimi et al., 2012). Briefly, under deep anesthesia (sevoflurane 3–4% in 100% O_2_; intravenous alfentanil 7–23 mg/kg/h), small craniotomies were made over the left and right motor cortex to permit epidural field potential recordings, and more caudally to permit pyramidal tract electrode placement. Stimulating electrodes were implanted into the pyramidal tract bilaterally using a double-angle approach (Soteropoulos and Baker, 2006). The initial stereotaxic target was 3 mm posterior and 10 mm ventral to the interaural line, 1.3–2 mm from the midline; electrodes were fixed at the location of lowest threshold to evoke an antidromic volley in the ipsilateral epidural recording from M1. A thermocoagulation probe (catalog #TC-0.7-2-200, Cosman Medical) was inserted into the left pyramidal tract rostral to the stimulating electrode (target coordinates for monkeys P and O, respectively: 3 mm posterior and 1 mm anterior, 10 and 6 mm ventral to the interaural line, 2 and 1.5 mm lateral to the midline). Again, the final depth was determined with reference to the threshold for evoking antidromic volleys. Repeated lesions (five and three for monkeys P and O, respectively) were made by raising the probe tip temperature to 60–75°C for 20 s, until the antidromic volley evoked from the pyramidal tract electrode (300 μA, 0.1 ms biphasic pulses) on the left side was abolished. All electrodes and the lesion probe were then removed, the wounds were repaired, and the animal was allowed to recover from anesthesia. Animals received a full program of postoperative care, including treatment with steroids to reduce brain edema (dexamethasone 0.15 mg/kg, i.v.), analgesia (0.01 mg/kg buprenorphine, i.v.; 0.1 mg/kg meloxicam orally daily), and prophylactic antibiotics (8 mg/kg cefovecin sodium, s.c. daily). Supportive therapy was provided as necessary in the immediate postoperative period, including intravenous fluids and tube feeding.

#### Surgical preparation

Seven and 8 months after the lesion (for monkeys O and P, respectively) or immediately after behavioral training was complete (for the control monkeys), each trained animal was implanted under general anesthesia (as above) and aseptic conditions with a headpiece to allow atraumatic head fixation, and a recording chamber to allow access to the pontomedullary reticular formation. A full program of postoperative analgesia (10 μg/kg buprenorphine; 5 mg/kg carprofen) and antibiotic care (co-amoxyiclav 140/35, 1.75 mg/kg clavulanic acid plus 7 mg/kg amoxycillin; 10 mg/kg cefalexin or 15 mg/kg amoxycillin) were delivered following the surgery in sequence or in conjunction depending on the response of the animal to the treatment.

#### Electrophysiological recordings

In daily sessions, multiple single neuron extracellular recordings were made from cells of the pontomedullary reticular formation using an Eckhorn microdrive loaded with glass-insulated platinum tetrodes (Thomas Recording). To minimize electrode deviation, sharpened guide tubes (Soteropoulos and Baker, 2006) were driven through the cortical dura up to the tentorium before searching for cells. Sites within the motor reticular formation were identified based on limb muscle responses to intracranial microstimulation through the recording electrodes. Such stimulation also revealed the locations of important brainstem landmarks such as the facial and abducens nucleus, and the superior colliculus, which allowed recording sites to be related to a published atlas (Martin and Bowden, 1996). Recordings were made from up to five electrodes simultaneously.

During recordings, spike waveforms (300 Hz to 10 kHz bandpass) and the local field potential (LFP; 1–100 Hz bandpass) were sampled continuously at 25,000 and 500 samples/s, respectively, from all four contacts of each tetrode and were stored to hard disc together with behavioral task markers. Off-line, spike occurrence times were discriminated from the raw waveforms using custom-written cluster-cutting routines (Getspike, S. N. Baker; SpikeLab, Dyball and Bhumbra, 2003).

#### Assessment of task-related modulation in discharge

Analysis of task-related modulation followed our previous work (Soteropoulos et al., 2012). A perievent time histogram (PETH) was compiled (10 ms bin width), averaging neuronal activity relative to the end of the second finger flexion or extension for monkeys D and R, or at the “pick” event for monkeys L, G, and P. The relative depth of modulation (RDM) was measured from the PETH as the difference between the maximal and minimal rates divided by the mean rate over the movement period (from 4 s before to 2 s after the end of the movement and from 2 s before to 2 s after the pick). A shuffling method determined whether the modulation was significantly different from zero. ISIs for each trial were shuffled randomly 1000 times; for each shuffle, the PETH was recalculated, and the peak modulation measured. If the modulation of the unshuffled PETH was >95% of the modulations after shuffling, the cell was assumed to be significantly modulated by that event (*p* < 0.05).

#### Calculation of features from extracellular records

The following features were extracted from the recordings of each cell.

##### Spike width.

**Figure 1.**
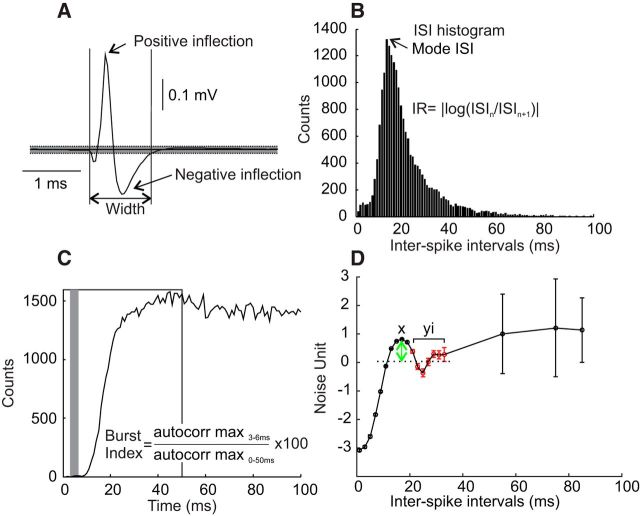
Measures used to characterize extracellular discharge. ***A***, Spike width. Horizontal lines show mean ± 3 SDs of baseline region before spike onset. Width was defined from the first and last crossings of these limits. The voltage at the spike positive and negative inflections were measured to calculate the normalized spike height. ***B***, ISI histogram, used to find the mode ISI and the IR index. ***C***, Autocorrelogram, used to calculate the burst index as the ratio between the maximum over 3–6 ms (gray box) to the maximal over 0–50 ms (white box). ***D***, The composite distance-to-threshold trajectory was computed by transformation of the ISI histogram (circles with error bars). The maximal peak was determined from the trajectory (*x*) and compared with the mean (dotted line) of manually selected neighboring data points on the right of the curve (*y_i_* in red), which allowed estimation of the peak height (green arrow).

For each cell, an average of the spike waveform was produced, triggered by the discriminated spike times, using the first 1000 available spikes (Fig. 1*A*). The baseline mean and SD values were calculated from a 1.5–2 ms section preceding the spike. The spike onset was detected as the first point above or below the baseline ±3 SDs. The offset of the spike was defined as the point where the waveform returned to within this window for at least 10 sample points. The spike width was measured as the time difference between the onset and the offset (Witham and Baker, 2007).

##### Normalized spike height.

Each spike was normalized by dividing its voltage by its peak-to-peak amplitude. Then the spikes were aligned relative to the occurrence time of their largest positive or negative inflection (Fig. 1*A*). The voltage at that time provided the normalized spike height and allowed the separation of spikes with a negative versus a positive peak. The values of the normalized spike height ranged from −1 to −0.5 and from 0.5 to 1 for spike shapes with a larger negative or positive inflection, respectively.

##### Mode interspike interval.

The ISI histogram was compiled across all available spikes, using 1 ms bins; the interval with the largest counts was taken as the mode ISI (Fig. 1*B*).

##### Irregularity.

Given a series of *N* interspike intervals *I_i_*, a measure of the spike train irregularity (IR; Davies et al., 2006), was calculated as follows:

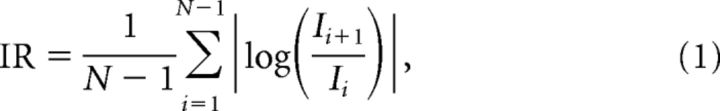
 where log is the natural logarithm, and |·| indicates the absolute value.

##### Burst index.

Following previous work (Barthó et al., 2004), the burst index was calculated as the maximal value of the autocorrelogram between 3 and 6 ms divided by the maximum value of the first 50 ms, expressed as a percentage (Fig. 1*C*):

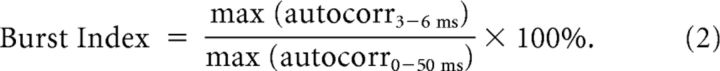


##### Peak height in distance-to-threshold trajectory.

The calculation of the distance-to-threshold trajectory used methods that have been described in detail previously (Wetmore and Baker, 2004; Witham and Baker, 2007) and will only be briefly summarized here. The objective was to reconstruct the trajectory of the membrane potential of the cell after firing an action potential, as it recovered from the afterspike refractory period and returned to just below threshold, ready to fire the next spike. Such measures are more usually determined from intracellular recording; the method that we used extracted an estimate of this trajectory from the statistics of extracellular spiking. Interspike intervals were first separated according to the prevailing firing rate, which was estimated from the surrounding intervals. ISI histograms were then compiled from intervals drawn from narrow ranges of firing rate and were used to compute interval death rate curves. The death rate is the probability that an interval will end within a given time bin, given that it has not already ended. The death rate was then transformed into the distance-to-threshold trajectory, using a monotonic transform determined on the basis of simulations of an integrate-and-fire model neuron responding to Gaussian input noise. Such distance-to-threshold trajectories were scaled in units of the SD of the membrane voltage noise. Trajectories from different firing rate ranges were combined by shifting vertically and averaging, yielding the composite distance-to-threshold trajectory (Fig. 1*D*). To determine whether the trajectory contained a significant peak (green arrow), the maximum of the trajectory (*x*) was compared with the mean of *N* adjacent values (*y_i_*) on the right. This region was manually chosen and represented a trough or a progressive decline of the trajectory after the maximal peak was reached. A *z*-score was calculated as follows:

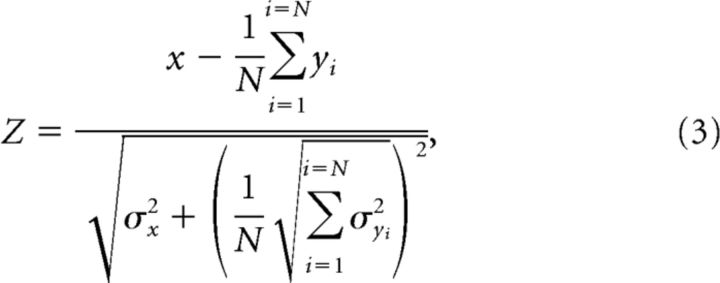
 where σ denotes the SE of the trajectory estimate calculated by the algorithm. If the value of *z* was 2.32, then we accepted that this cell had a significant peak in its trajectory (*p* < 0.01, based on standard normal distribution, one-tailed test).

#### Cluster analysis

Six measures were made from each cell as follows: mode ISI, IR, burst index, spike width, peak in distance-to-threshold trajectory, and normalized spike height. These measures were assembled into a table across all cells recorded from the control monkeys, and each value was then transformed by subtracting the mean and dividing by the SD, forming *z*-scores. To estimate in an objective way how many clusters were present in the data, we used three different clustering algorithms provided by the evacluster routine in Matlab (kmeans, linkage, and gmdistribution). We selected these algorithms because they represent three different methods to calculate the optimal number of clusters using the Calinski–Harabasz (variance ratio) criterion. The kmeans algorithm selects observations at random from the data to be the putative cluster centroids; other data points are then assigned to the closest cluster based on Euclidean distance. Points are moved between clusters if such a move reduces the variance ratio, until no further moves are possible. The linkage algorithm creates a hierarchical tree from the data based on pairwise distance; clustering is determined by the threshold at which this tree is cut. The gmdistribution method fits the data to a Gaussian mixture model, and assigns each observation to the component with the highest posterior probability. To test the stability of the cluster evaluation methods, the kmeans and gmdistribution methods were run 1000 times with different initial conditions. This was unnecessary for the linkage algorithm, as it produces deterministic results with no requirement for random initial conditions.

#### Identification of reticulospinal cells in recordings under anesthesia

Extracellular activity from the reticular formation was recorded from five female macaque monkeys under terminal anesthesia, and cells were identified as reticulospinal if they responded antidromically to stimulation of the ventrolateral funiculus of the spinal cord around the C4–C5 level. Full methods for similar recordings have been published previously (Fisher et al., 2012). Responses were taken as antidromic if they responded to spinal stimulation at low jitter (<0.1 ms), with a sharp threshold for all-or-none activation. Low spontaneous firing rates often precluded the performance of a collision test, but this was performed successfully in a subset of the cells (Baker et al., 1999). The spike widths were calculated using the method described above.

## Results

### Clustering extracellular features reveals evidence for four classes of reticular neurons

**Figure 2.**
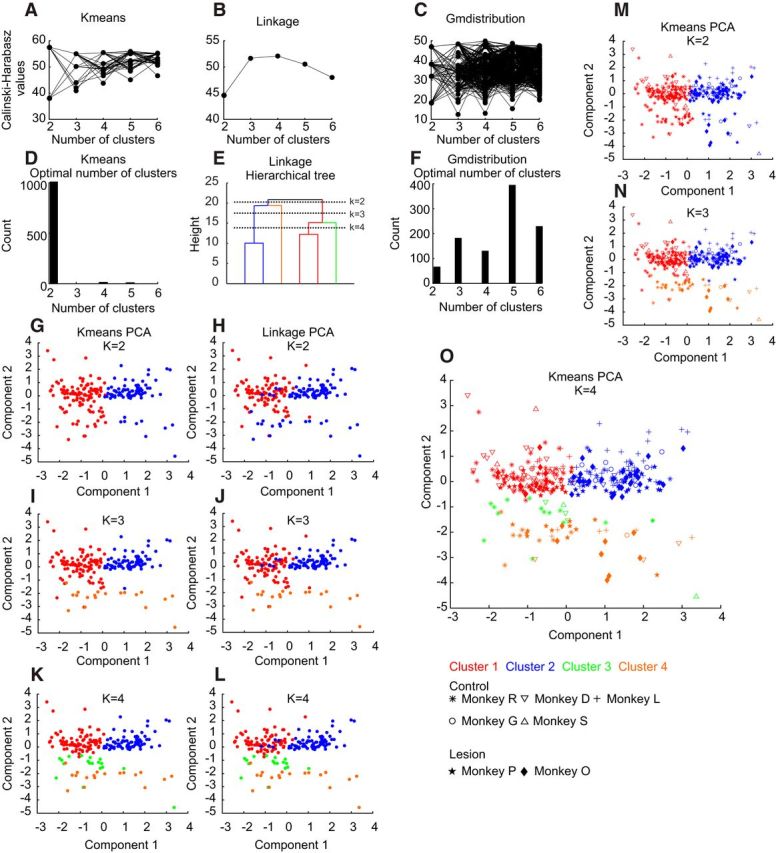
Cluster analysis. ***A–C***, Evaluation of the optimal number of data clusters using kmeans, linkage, and gmdistribution algorithms with the Calinski–Harabasz criterion (evacluster routine in Matlab). Overlain results from 1000 repeats with different starting conditions are shown. ***D***, Histogram of the solutions provided by the kmeans algorithm. ***E***, Hierarchical cluster tree as provided by the linkage algorithm (method set to “ward”: inner squared distance, Matlab). The links between the clusters are represented with their respective heights (distances). The colors of the lines correspond to the different clusters and can be traced from higher clusters, for each defined maximum number of clusters (*k*). ***F***, Histogram of the solutions provided by the gmdistribution algorithm. ***G***, ***I***, ***K***, The principal component projection of the cell measurements onto the two first components. Each point shows measures from one cell, using the kmeans method with the optimal number fixed to 2, 3, and 4 clusters, respectively. ***H***, ***J***, ***L***, same as ***G***, ***I***, and ***K*** but using the linkage and cluster algorithms (Matlab). ***M–O***, Same as ***G***, ***I***, and ***K***, but different symbols mark results from different animals, with the addition of animals after the pyramidal tract lesion (filled symbols). Colors indicate the clusters to which each point was assigned.

The kmeans method (Fig. 2*A*) was very stable and gave an optimal number of clusters of two for 99.4% of the 1000 iterations (Fig. 2*D*). The linkage method (Fig. 2*B*) gave an optimal cluster number, which was 4. In contrast, the Gaussian mixture distribution algorithm was noisier (Fig. 2*C*): for 40% of iterations, the optimal cluster number was 5, but optimal cluster numbers of 6, 3, 4, and 2 were generated in 23%, 18%, 13%, and 7% of the iterations, respectively (Fig. 2*F*).

The linkage method creates a hierarchical tree where the clusters are joined at different levels; the heights of the links represents the distances between clusters. This method allows us to track the origin of each new cluster from the clusters at a higher level. By determining a maximum number of clusters (*k*; Fig. 2*E*, dotted line), the descending branches of the tree are pruned back until this number remains. Figure 2*E* shows that for *k* = 3 the orange cluster originates from the blue cluster. Then at *k* = 4 (the optimal number defined by linkage), the green cluster originates from the red cluster.

The data were subjected to principal component analysis, allowing them to be visualized as a scatter plot of the first two principal components, which accounted for 51.5% of the total variance. In Figure 2, results from the kmeans method (Fig. 2*G*,*I*,*K*) and linkage method (Fig. 2*H*,*J*,*L*) are shown. For two clusters, the assignment of points into the red and blue clusters was similar, independent of the clustering method (Fig. 2*G*,*H*). The main difference was for points with negative component 2, which were assigned to the red cluster for kmeans, but to the blue cluster for linkage. Interestingly, these points were reliably assigned to the third cluster by both methods when the cluster number was set to 3 (Fig. 2*I*,*J*). The fact that a conflict between methods was resolved by increasing the cluster number provides confidence that at least three clusters are present. The addition of a fourth cluster also led to a consistent assignment of points to this new cluster by both methods (Fig. 2*K*,*L*).

Different formal methods to determine the cluster number suggested *k* = 2, 4, or 5 clusters (Fig. 2*A–F*), although with some uncertainty. We excluded using 2 clusters, as the different methods did not agree on the assignment of points (Fig. 2*G*,*H*), which was improved with more clusters. We consider this a signature of choosing a cluster number that is too low. Using five clusters would have led to low occupancy in some clusters, which would make it hard to draw conclusions on intercluster differences. We therefore chose to use 4 clusters for the remainder of this article. We recognize that this is to some extent an arbitrary decision. We do not claim that this number is definitive; rather, that by using it we can make progress in understanding the neurobiology of the reticular formation. This issue is considered in more detail in the Discussion.

The colors used to mark the 4 clusters in Figure 2 are used throughout the remaining figures of this article. Table 1 presents the numbers of cells assigned to each cluster across animals. The majority of cells (82%) was divided between clusters 1 and 2, and each monkey contributed data to these clusters. Clusters 3 and 4 had lower occupancy; each animal contributed cells to at least one of these clusters. The consistency across animals argues against the underlying variation, reflecting differences between animals, or in the precise location of recordings that could have differed systematically across the animals.

**Table 1.** Number of cells per cluster for each monkey

	Cluster 1	Cluster 2	Cluster 3	Cluster 4
Control animals				
Monkey R	35	13	13	8
Monkey D	29	8	3	3
Monkey L	11	39	0	6
Monkey G	5	11	0	1
Monkey S	7	3	2	0
Totals	87 (44%)	74 (38%)	18 (9%)	18 (9%)
Lesioned animals				
Monkey P	37	33	1	13
Monkey O	8	26	0	8
Totals	45 (36%)	59 (47%)	1 (<1%)	21 (17%)

Each feature from the animals after recovery from lesion was normalized relative to the mean and SD of the corresponding measure in control animals. These features were then used to determine cluster membership, using the same cluster centers as for controls. The projections into the first two components are shown for all of the different monkeys for an optimal number of clusters of 2 (Fig. 2*M*), 3 (Fig. 2*N*), or 4 (Fig. 2*O*).

### Distribution of features across clusters

In Table 2, the mean and SD values of the six features (mode ISI, IR, spike width, burst index, the afterspike distance-to-threshold peak height, and the normalized peak height) that were used to cluster the data are presented. The cluster centers, expressed in standardized units (zero mean, unit SD), indicate how the specific measures varied between clusters. Table 2 will allow future studies to allocate neurons to clusters in the same way as we have done.

**Table 2.** Mean and SD of measures taken from the reticular formation recordings

	Mean	SD	Cluster centers (*z*-score units)			
			Cluster 1	Cluster 2	Cluster 3	Cluster 4
Mode ISI (ms)	27.917	21.642	0.310	−0.161	−0.421	−0.425
Irregularity	0.789	0.360	−0.629	0.893	−0.519	0.005
Spike width (ms)	2.026	1.024	0.273	−0.300	0.118	−0.239
Burst index	36.264	31.365	−0.709	0.917	−0.346	0.119
DTT peak height (noise units)	0.069	0.196	−0.343	−0.271	2.565	0.142
Normalized spike height	0.554	0.434	0.664	0.730	0.605	−0.722

Each value was transformed to a *z*-score by subtracting the mean and dividing by the SD; the centers of each cluster in this normalized space are presented.

**Figure 3.**
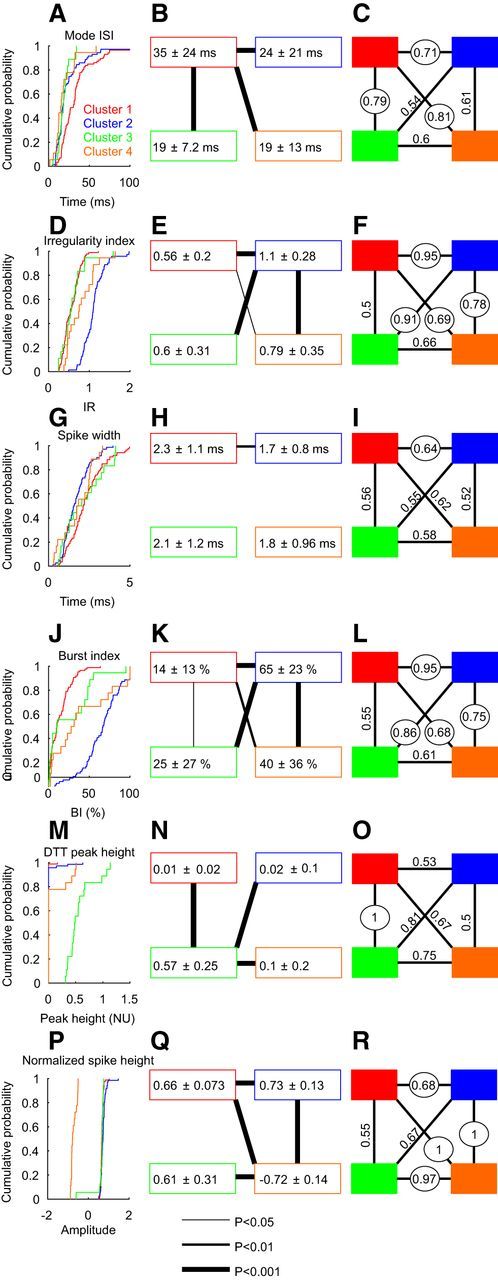
Distributions of features across clusters. Each row relates to a different feature. Left column shows the cumulative distribution of the measure, shown separately for each cluster. Middle column gives the mean and SD values of each measure. The lines between each pair of boxes indicate a significant difference between those clusters (Kolmogorv–Smirnov test; thicker lines indicate lower significance levels). Right column presents the area under the receiver operating characteristic curve, marked on the lines joining a cluster pair. Numbers are circled where they were significantly different from chance level (0.5). ***A–C***, Mode ISI. ***D–F***, IR. ***G–I***, Spike width. ***J–L***, Burst index. ***M–O***, Distance-to-threshold trajectory peak height. ***P***, ***Q***, ***R***, Normalized spike height. Colors denote results from different clusters, using the color code shown in ***A***.

Figure 3 illustrates how the six different features used for clustering were distributed. In the left column, the cumulative probability distribution of each measure is shown. The middle column reports the mean and SD values of these distributions. The significance or otherwise of pairwise comparisons is indicated by the width of the lines between boxes in this column. It was often the case that differences were seen mainly for comparisons with one of the clusters, although the identity of this cluster varied depending on the measure used. For example, the mode ISI (Fig. 3*A–C*) was significantly larger for cluster 1 than for clusters 2, 3, and 4 (*p* < 0.01), but clusters 2 and 3 did not differ significantly from each other. Cluster 2 differed from all of the others for irregularity, cluster 3 for distance-to-threshold trajectory peak height, and cluster 4 for normalized spike height. This strongly suggests that there is an underlying basis for division of the data into four clusters.

Even where significant differences were observed, distributions were often heavily overlapping. One way to measure how well a given measurement can discriminate between two populations is to compute the receiver operating characteristic (ROC) curve (Green and Swets, 1966) and to measure the area under this curve (AUC). If two populations have identical distributions, the AUC will equal 0.5. An AUC value of 1.0 indicates perfect separation in the distribution. The right column of Figure 3 shows AUC values calculated between pairs of clusters on each measure; the values circled were significantly different from 0.5, as estimated using a Monte Carlo resampling method.

**Figure 4.**
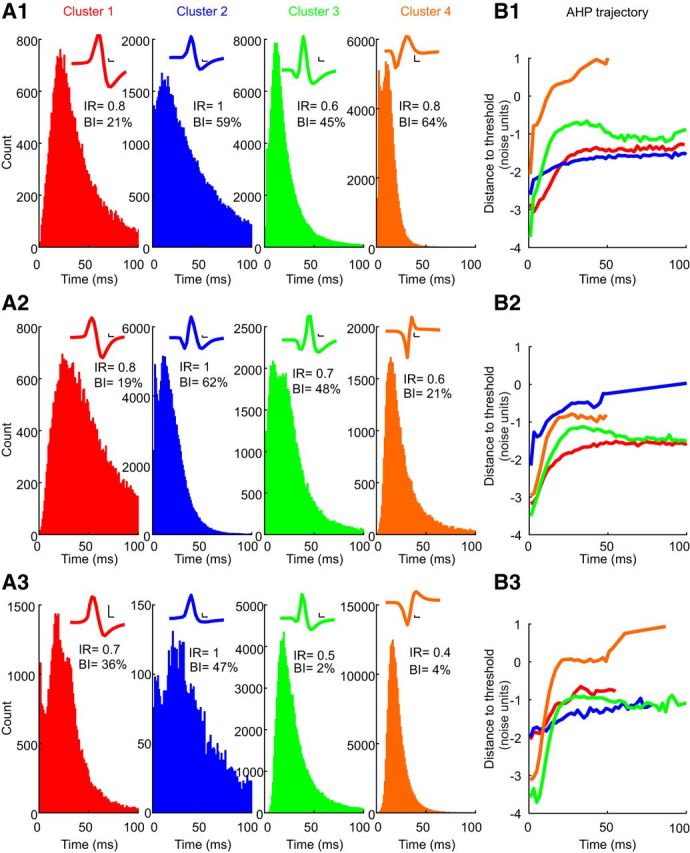
Example results from the three most representative cells from each cluster (indicated by colors). Columns show the following: ***A***, ISI histograms with overlain spike waveforms. Calibration: 0.1 ms, 0.2 mV. ***B***, Distance-to-threshold trajectories. Rows show the cells closest (1), second closest (2), and third closest (3) to their respective cluster centers.

Figure 4 presents the three most representative cells for each cluster—these are the cells closest, second closest, and third closest to the cluster center (top, middle, and bottom rows, respectively). These cells serve again to emphasize not only the differences between clusters, but also the overlapping nature of the distributions. For example, the cell closest to the center of cluster 4 (Fig. 4*A*, orange) exhibits a high bursting index that does not represent that cluster at the population level (Fig. 3*J*). However, these cells do represent the fundamental characteristics of their cluster population: cluster 1 cells have large mode ISIs; cluster 2 cells show a high irregularity and bursting indices; cluster 3 cells show AHP peaks; and cluster 4 cells have spike waveforms with negatives peaks. Clusters 1 and 3 have no peak in their distance-to-threshold trajectory, leading to irregular firing with a high burst index.

### Lack of spatial separation of cell classes

**Figure 5.**
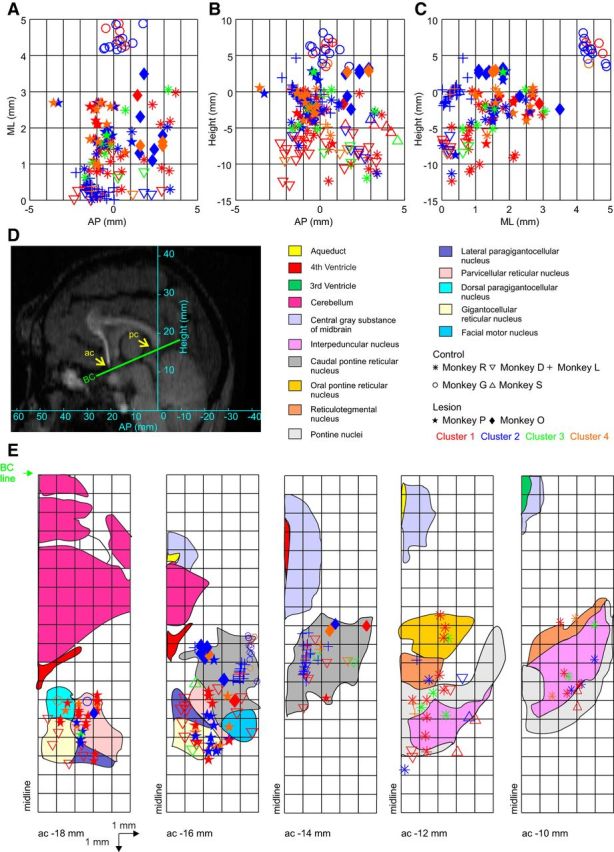
Estimated location of the recorded cells. Recording sites are projected into three stereotaxic planes, with zero corresponding to the midline at the interaural line. Different symbols indicate different animals; colors indicate the assignment of cells to the four clusters. ***A***, Horizontal plane. ***B***, Parasagittal plane. ***C***, Coronal plane. ***D***, Parasagittal MRI slice from monkey R showing the bicommissural (BC) line between anterior commissure (ac) and posterior commissure (pc). ***E***, Penetrations rotated into the BC coordinates and were overlain on an atlas template with the representation of different reticular nuclei and landmarks.

Figure 5 presents the calculated locations of all cells recorded as projections onto the canonical stereotaxic planes (Fig. 5*A–C*). These have been estimated using the location of the recording chambers, which were measured in the implant surgeries, and the location and angles of the electrodes relative to the chambers as measured during the recording sessions. It is clear that there was no spatial separation of the different cell clusters, but rather that recordings from all clusters were obtained throughout the region sampled. Recordings from one animal, monkey G, were noticeably more lateral than in the others, but cells were still classified into three of the four clusters in this monkey. To reconstruct the penetrations in the reticular formation, the stereotaxic coordinates of the anterior and posterior commissures were identified on the MRI for each monkey (Fig. 5*D*). The penetration sites in the reticular formation were then adjusted to bicommissural coordinates and projected (Fig. 5*E*) on an atlas template (Martin and Bowden, 1996). Based on the location relative to the abducens and facial nuclei, these recordings are likely to span all of the major reticular nuclei, including the nucleus giganotcellularis, pontine reticular nucleus pars caudalis, and par oralis.

### Postspike distance-to-threshold trajectories

**Figure 6.**
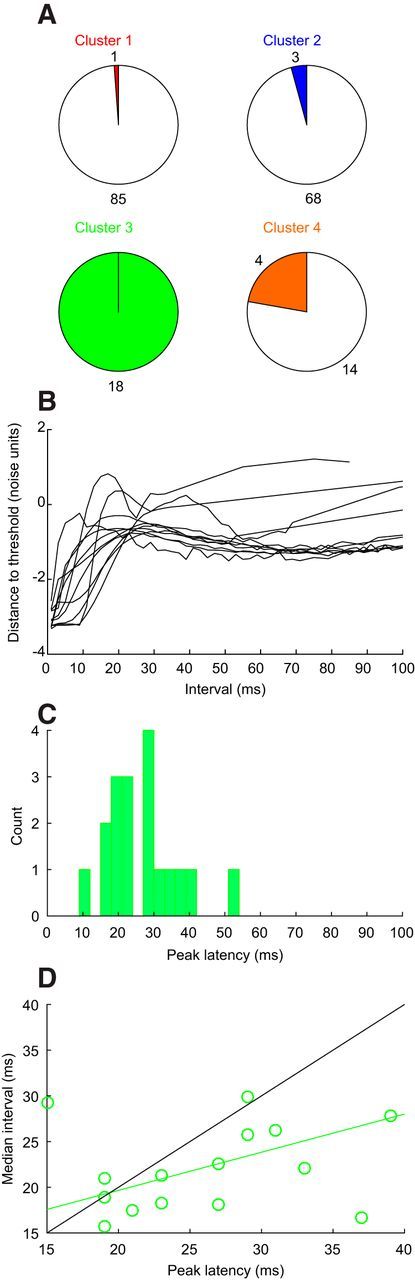
Peaks in distance-to-threshold trajectory. ***A***, Pie chart representation of the proportion of cells with a significant peak (colored segment) in their distance-to-threshold trajectory, for the different clusters. ***B***, Overlain distance-to-threshold trajectories for the 10 cells from cluster 3 with largest peaks. ***C***, Histogram of the time of the peak. ***D***, Scatter plot of median interspike interval vs peak latency. Green line, least square regression fit; black line, identity. For ***C*** and ***D***, only results from cells in cluster 3 with peaks significantly larger than zero have been included.

As illustrated in Figure 1*D*, we derived an estimate of the afterspike DTT from the interspike interval distribution of each cell. The height of the peak in the DTT was used in the cluster analysis, regardless of whether it was significantly different from zero. Cluster 3 exclusively contained cells with significant DTT peaks and clearly had larger peaks than the other three clusters (Fig. 3*M–O*). Figure 6 presents further analysis of the peaks in DTT. Aside from cluster 3, a low proportion of cells in other clusters also had peaks that reached our significance criterion (Fig. 6*A*). The significant peak heights in cluster 3 (Fig. 6*B*) were relatively large (0.49 ± 0.07 noise units), and their latency was distributed around a wide range of timing (Fig. 6*C*, peak latency 25.0 ± 2.9 ms, median ± SE of the median). There was a strong correlation (Fig. 6*D*) between the peak latency and median interspike interval (*r*^2^ = 0.42, *p* < 0.01), although the slope of the linear regression line (Fig. 6*C*, green; 0.42; 95% confidence limits, 0.16–0.67) was significantly smaller than the identity line (Fig. 6*C*, black), suggesting that other factors tended to reduce the variation in firing rate across this cluster rather than it only being determined by the timing of the DTT peak.

### Local circuit connections

**Figure 7.**
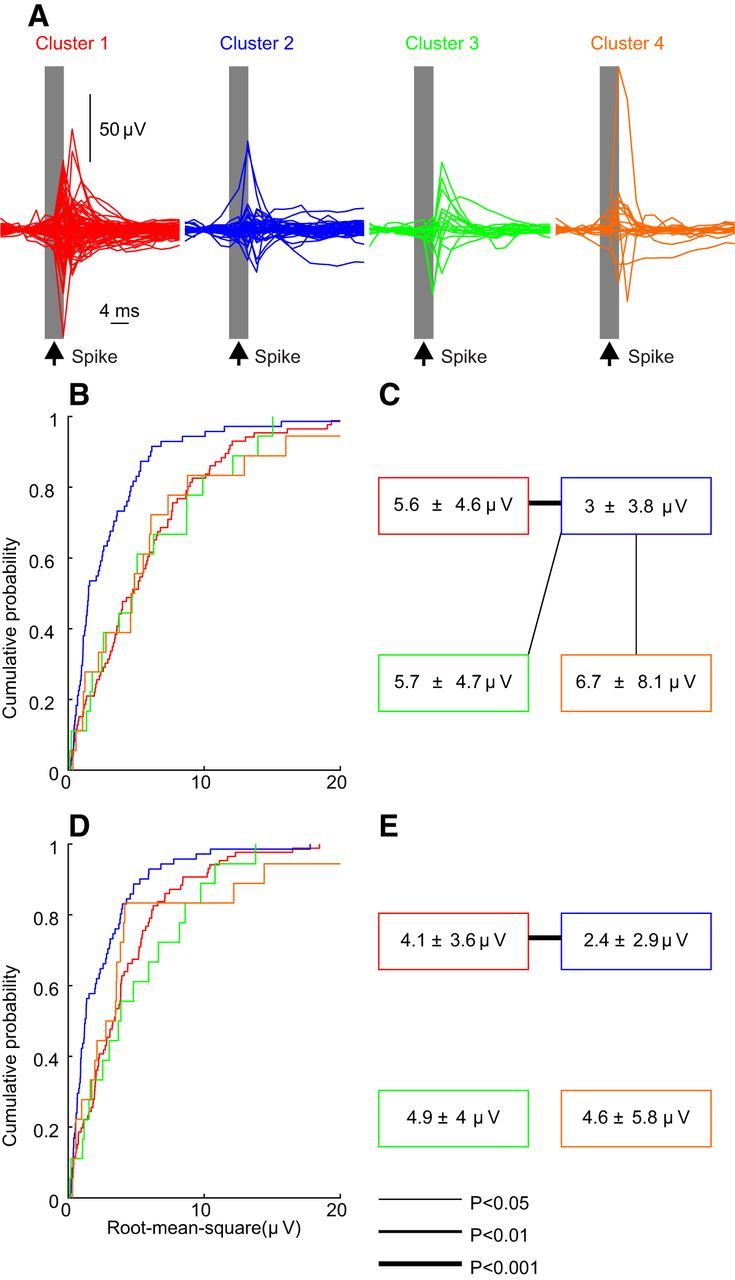
Spike-triggered average of LFP recorded on the same electrode as the spike. A, Overlain spike-triggered averages from all cells in control animals, separated by cluster. ***B***, Distribution of the root mean square amplitude of the average, plotted as a cumulative probability distribution separated by cluster. ***C***, Mean and SD of the amplitudes in each cluster. Lines joining the boxes indicate significant (Kolmogorov–Smirnov test) pairwise differences. ***D***, ***E***, Similar plots as in ***B*** and ***C***, except that amplitudes are computed excluding the period 2 ms on either side of the spike time (shaded gray in ***A***).

To assess the impact of a spike on the local network, we performed spike-triggered averaging of the LFP recorded on the same electrode. We expect that the reticular formation will have an extensive local network of synaptic connections between cells, and these averages will reflect such connections. They will also contain contributions from intrinsic conductance within the cell generating the triggering spike, and could also contain contributions from gap junctional coupling between cells if these existed. Such averages are shown in Figure 7*A*, overlain for all cells within one cluster. Both positive and negative deflections were seen within each cluster. These may reflect a mixture of excitatory and inhibitory effects, but equally could reflect random variation in the location of the recording electrode relative to local cells generating the currents.

To provide a gross measure of the size of these effects, we computed the root mean square amplitude of the traces over the time interval illustrated (12 ms before to 28 ms after the triggering spike). The cumulative distribution of this measure is illustrated in Figure 7*B*, and to the right are shown the results of pairwise comparisons between clusters (Fig. 7*C*). The effects from cluster 2 were significantly smaller than those in the three other clusters. However, spikes from cluster 2 were generally of lower amplitude (data not shown). It is known that low-frequency components of spikes can contaminate the LFP (Waldert et al., 2013), which for cortical recordings typically leads to a brief artifact on spike-triggered averages of LFP measured from the same electrode as the triggering spike (Baker et al., 1997). To exclude the possibility that the differences shown in Figure 7*B* and *C*, could reflect differences in spike height between clusters, we recomputed the root mean square values, excluding the region from 2 ms before to 2 ms after the spike (Fig. 7*A*, shaded gray area). This resulted in the cumulative distribution plotted in Figure 7*D*, which had similar differences between cluster 2 and the other three clusters, as shown when using the whole trace (Fig. 7*D*). However, only the root mean square amplitude between cluster 2 and cluster 1 remained significantly different.

### Task-dependent rate modulation

The majority of the cells recorded in all four clusters showed significant task-dependent modulation of firing rate with both the flexion and extension phases of the slow finger movement task, and the food retrieval task (in clusters 1, 2, 3, and 4, respectively: flexion: 95%, 74%, 87%, and 100%; extension: 91%, 89%, 81%, and 100%; food picking: 93%, 96%, unavailable, and 100%; no cell from cluster 3 was recorded during the pick).

**Figure 8.**
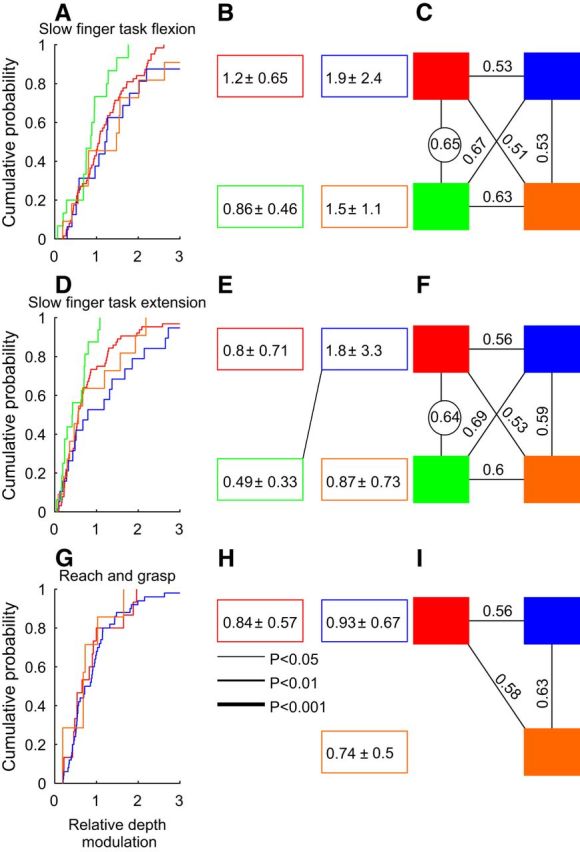
Task-dependent modulation of firing rate. ***A***, Cumulative probability distribution of RDM (see Materials and Methods) for cells in each cluster, for finger flexion trials in monkeys R and D. ***B***, Mean and SD of RDM for each cluster. ***C***, Area under the receiver operating characteristic curve for pairwise cluster comparisons. Circled numbers indicate values significantly different from chance level (0.5). ***D–F***, Similar display as for ***A–C***, except for extension trials in monkeys R and D. ***G–I***, Similar to ***A–C***, but for the reach-and-grasp task in monkeys L and G. The line in E indicates a significant difference between clusters (Kolmogorov–Smirnov test). No cell was recorded from cluster 3 during the reach-and-grasp task.

Figure 8 presents the distribution of the RDM as cumulative distributions, pairwise comparisons, and the AUC of the ROC curves, following the same display convention as in Figure 3. Cluster 3 showed smaller depth modulations than the three other clusters during flexion, with the ROC analysis showing a clear separation between the curves of clusters 3 and 1 (Fig. 8*A–C*). The ROC analysis also reached the significance limit showing the separation between these two clusters during extension. The relative depth modulation of cluster 3 was also significantly smaller than that of cluster 2 (Fig. 8*D–F*). Note that for the food retrieval task no cell was available for analysis in cluster 3, so we could not compare this cluster to the other three clusters that showed similar depth modulations. However, a consistent finding was the lower task-related modulation of firing in cluster 3, during both flexion and extension.

### Antidromically identified reticulospinal tract neurons

**Figure 9.**
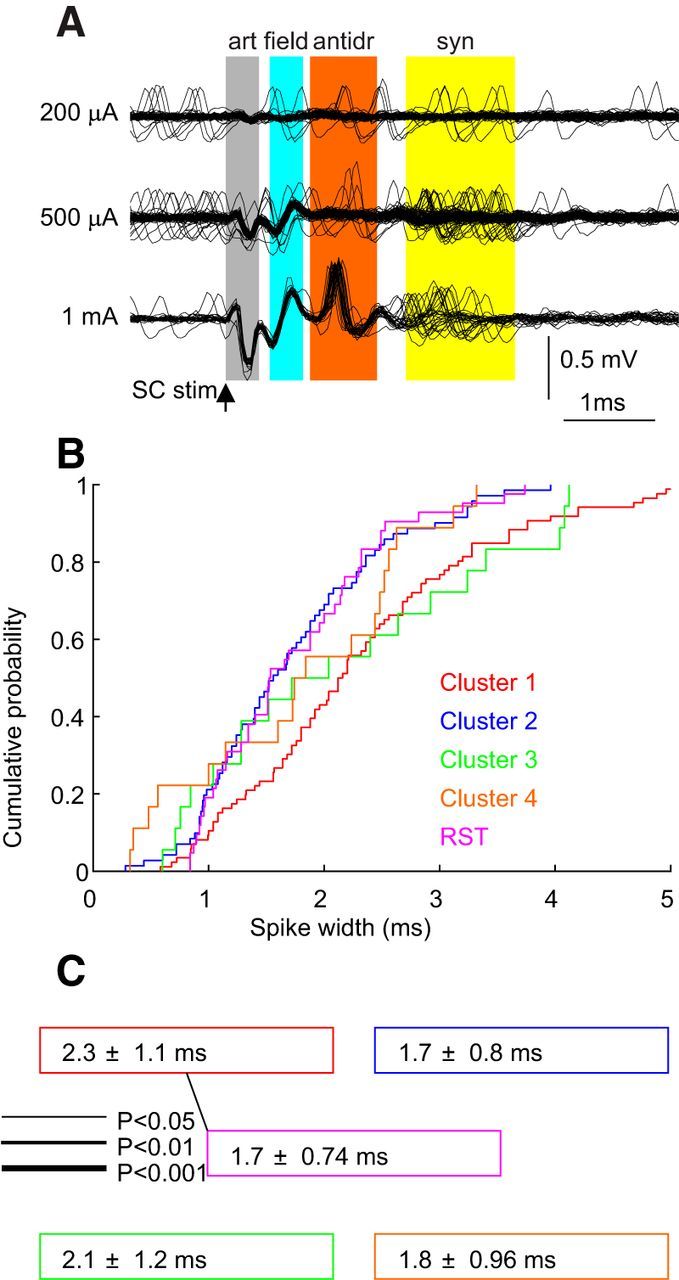
Measurements from identified reticulospinal cells. ***A***, Example cell identification. Traces show recordings from the reticular formation following spinal stimulation (C6 level) at different intensities (arrow). After the stimulus artifact (“art”; gray shading) came a field response (“field”; blue shading), identified by its graded increase with rising stimulus intensity. At 1 mA stimulation, an all-or-nothing antidromic spike appeared at 1.1 ms latency (“antidr”; orange). Longer-latency spikes were likely to be synaptic responses, based on their high jitter (“syn”; yellow shading). ***B***, Distribution of spike widths for antidromically identified RST cells (purple) overlain on the four clusters identified in this article (data replicated from Fig. 3*G*). ***C***, Mean ± SD of spike widths for each cell class, using the same colors as in ***B***. Lines show pairwise comparisons between RST cells and clusters with significantly different distributions (Kolmogorov–Smirnov test).

During the recordings in awake monkeys, we did not have stimulating electrodes implanted in the spinal cord and, hence, were not able to identify reticulospinal neurons by antidromic activation. However, a limited number of cells were antidromically identified in anesthetized animals. One example of such identification is illustrated in Figure 9*A*. At a low stimulus intensity (200 μA), no consistent response was observed in the extracellular record. When the intensity was raised to 500 μA, a small short-latency field potential appeared (labeled “field”); this was distinguished from a spike by its graded growth with increasing intensity (compare with trace at 1 mA). At a longer latency, a neural spike responded on some sweeps, and with a variable latency (labeled “syn”). This response was synaptically mediated; we cannot be certain which pathway mediated this. At a stimulus intensity of 1 mA, a reliable response appeared with low jitter; this was an antidromic response (labeled “antidr”).

The conduction velocity (21.9 ± 6.3 m/s) was calculated for 21 identified reticulospinal cells by dividing the distance between the stimulating electrodes and obex by the latency of the antidromic spike.

Many of the measures that we have used in our main dataset were either impossible in the anesthetized animals (e.g., task-related firing) or likely to be altered by the anesthesia (e.g., mode ISI, IR). However, it was possible to determine the spike width of the antidromically identified units. We might expect that this would not be altered by the anesthetic mixture used. Small differences could occur between antidromic and orthodromic spikes, although these are unlikely materially to change our measurements as we simply use the spike width, rather than a detailed metric of action potential shape. Figure 9*B* shows the cumulative probability distribution of the 42 identified reticulospinal cells, overlain on the four clusters identified from the awake recordings. The spike widths of the reticulospinal cells were significantly smaller than spike widths of cluster 1 (*p* < 0.05). No difference was observed between the spike widths of reticulospinal cells and clusters 2, 3, and 4 (Fig. 9*C*); in fact, the distributions for identified reticulospinal cells and cluster 2 overlay very closely.

### Characterization of pyramidal tract lesion

**Figure 10.**
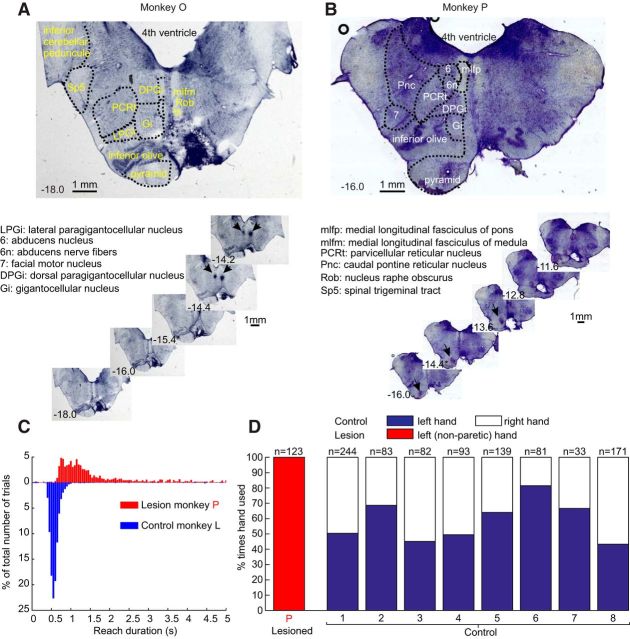
Characterization of pyramidal tract lesions. ***A***, ***B***, Histological sections of the brainstems from the two monkeys. Numbers in the bottom left of each photomicrograph are the estimated rostrocaudal location of each section, using the coordinates of Martin and Bowden (1996), which were also used to identify the nuclei shown in outline on the representative sections at the top. Arrows in the small photomicrographs indicate the location of the tips of electrodes in the medial longitudinal fasciculus in monkey O and the nonlesioned pyramidal tract in monkey P, which were used for other unrelated studies and were marked with electrolytic lesions just before the animal was killed. The section with rostrocaudal estimated location marked with an asterisk is the point with the largest lesion extent. ***C***, Distribution of times taken for food retrieval, for one lesioned and one control animal. The time is measured from the moment the clear plastic door dropped, to the moment the infrared beams surrounding the food well were broken. *N* = 3226 and 6227 trials, respectively, for lesioned and control animals. ***D***, Hand use preference. Bars show the fraction of times that each hand was used to retrieve food during foraging behavior in the home cage, measured from a video recording.

Two monkeys were subjected to a unilateral pyramidal tract lesion at the level of the medulla, allowed to recover for 1 year, and then neural activity was recorded from the reticular formation contralateral to the lesioned pyramid (ipsilateral to the paretic hand). Postmortem histological analysis verified the extent of the lesions (Fig. 10*A*,*B*).

In monkey O (Fig. 10*A*), the lesion had its greatest extent at the level of the inferior olive (Fig. 10*A*, bottom, section labeled −15.4). The lesion ablated completely the left pyramid, the left inferior olive, parts of left reticular formation, and parts of the left medial longitudinal fasciculus. The lesion spread to the contralateral side, where it also affected some part of the inferior olive. In monkey P (Fig. 10*B*), the lesion was more rostral and more restricted than in monkey O. It ablated a major part of the left pyramid, but at its greatest extent (Fig. 10*B*, section labeled −14.4), some fibers seem to have been spared on the lateral edge of the tract.

The time course of recovery in these two animals was similar to that in our previous report (Zaaimi et al., 2012). Immediately following the lesion surgery, animals showed a flaccid paralysis of the contralateral limbs. Their initial substantial disability necessitated single housing in a small recovery cage and a high level of care. Although no formal program of physiotherapy was provided, the limbs on the affected side were regularly mobilized to prevent joint contractures. After 11 d and only 1 d (for Monkeys O and P, respectively), the gross motor function of the animals was sufficiently well recovered to return safely to a larger holding cage with high-level perches. At 15 and 2 d after lesioning, recovery was sufficient to return to the large pen that formed the normal accommodation of the animals, and to rejoin the unlesioned cage mate with whom the monkeys had been housed before the surgery. These pens contained a range of items to promote locomotion, climbing, and foraging. This enriched and social environment provided a powerful and continual stimulus to functional recovery. The monkeys could climb, jump, and walk, in all respects showing a recovery similar to that in previous reports (Lawrence and Kuypers, 1968; Chapman and Wiesendanger, 1982; Lemon et al., 2012; Zaaimi et al., 2012).

Monkey P was chaired and retrained on the food retrieval task 17 d after the lesion. By day 33, this animal still had a poor grip, but for highly motivational rewards could successfully pull the lever and retrieve the food from the well. After 3 months of retraining, the monkey was performing the task relatively normally, although movements were slowed. Figure 10*C* presents the distribution of reach times in this animal during sessions in which cells were recorded. Reach time was measured between the moment the door dropped to expose the food well and when the hand broke the infrared beams surrounding the well. Reach duration was significantly slower in lesioned monkey P compared with control monkey L (1.32 ± 0.24 s vs 0.56 ± 0.01 s, median ± SE of the median; Wilcoxon rank-sum test, *p* < 0.001).

To provide a further assessment of hand function during natural behavior, monkey P was filmed for 45 min in the home cage while foraging for food (a mixed blend of cereals, dried fruits, peanuts, raisins, and seeds) scattered among the sawdust litter. The number of times the left or right hand was used to pick up the food was counted; a similar measurement was made for eight unlesioned monkeys (age range, 3.6–5.8 years; weight range, 6.0–8.5 kg) also housed in our laboratory around the same time. Monkey P never voluntarily used the more affected (right) side to retrieve food. By contrast, although control animals showed a varying degree of hand preference (maximum, 81%), in all cases performance was bilateral (Fig. 10*D*).

Monkey O showed a slower recovery, with a more marked residual deficit. This animal did not recover the ability to perform the reach-and-grasp task. Although the fingers could flex sufficiently to hold food if placed within the hand, independent finger movements were entirely lacking. When presented with a Klüver board, even large food morsels could not be retrieved.

### Changes in reticular formation activity after pyramidal tract lesion

Neural recordings from the reticular formation were made 370–403 d after lesioning in monkey P, and 363–423 d after lesioning in monkey O. All measures as described in Figure 1 were made from discriminated single units, and cells were assigned to one of the four clusters, depending on their proximity to each cluster center. To ensure that a direct comparison could be made between lesioned and control animals, clusters were not recomputed for the lesioned data, but rather the cell measurements were simply compared with the cluster centers computed from the control animals. The number of cells allocated to each cluster in the lesioned animals is given in Table 1; this distribution was not significantly different from that observed in the control animals (*p* > 0.05; χ^2^ test), except for the number of cells in cluster 3, which dramatically decreased after the lesion (9% vs 0.8%; *p* < 0.01, χ^2^ test).

**Figure 11.**
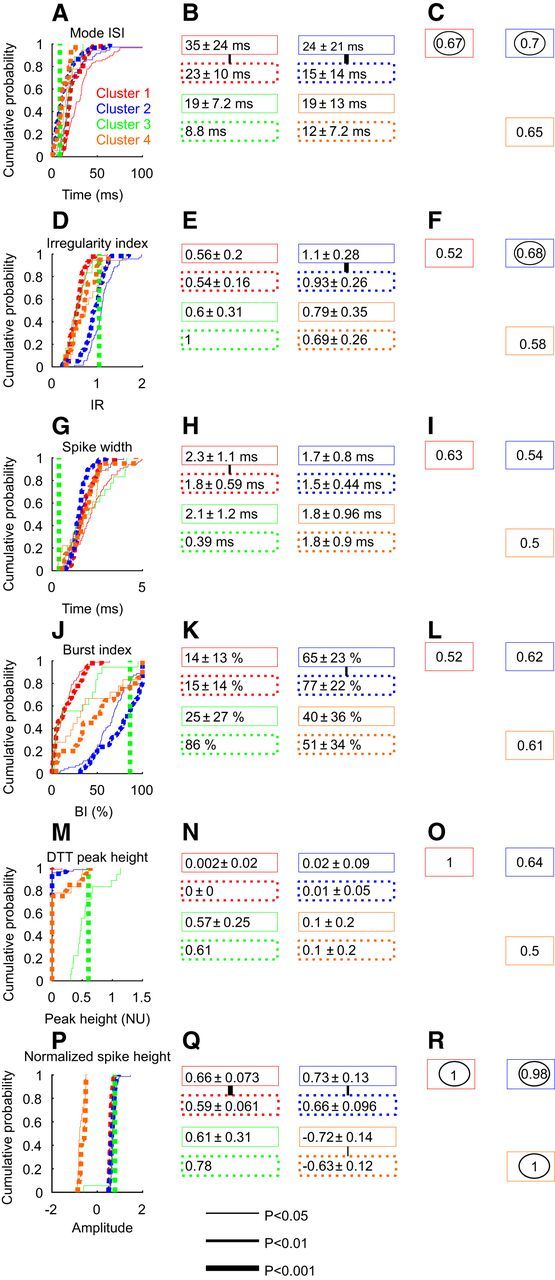
Distribution of the spiking features in cells recorded from control and lesioned monkeys. Display conventions are similar to those in Figure 3. The left column shows the cumulative probability distribution of the spike measures. Different colors present data from different clusters; solid lines indicate data from control; and dotted lines indicate data from lesioned monkeys. The middle column shows mean ± SD values of measures for each cluster, again with control and lesioned monkeys presented separately by solid and dashed boxes. Lines between control and lesion data indicate a significant difference (Kolmogorv–Smirnov test). The right column shows the area under the receiver operating characteristic curve for each measure, comparing control and lesion data. Numbers are circled where they are significantly above the chance level (0.5). Rows indicate results for different measurements. ***A***–***C***, Mode ISI. ***D–F***, Irregularity. ***G–I***, Spike width. ***J–L***, Burst index. ***M–O***, Peak height in the estimated DTT. ***P–R***, Normalized spike height.

Figure 11 presents the distribution of the measurements from control and lesioned animals for each cluster. Cluster 2 showed most of the significant changes after the lesioning. This cluster showed a significant decrease in the mode ISI (Fig. 11*A*,*B*), a change confirmed by the ROC analysis (Fig. 11*C*). Only cluster 2 showed a significant decrease in its irregularity index (Fig. 11*D*,*E*), which was sufficient to permit some separation between control and lesioned animals on the ROC analysis (Fig. 11*F*). Cluster 2 showed a significant increase in its burst index (Fig. 11*J*,*K*), which was not reflected by a significant separation in the ROC analysis (Fig. 11*L*). Cluster 1 showed a decrease in the mode ISI (Fig. 11*B*) and in the spike width (Fig. 11*H*); only the former reached significance in the ROC analysis (Fig. 11*C*,*I*). Only one cell was assigned to cluster 3 after the lesioning, which made it impossible to analyze the after-lesion variance of this cluster. No major change was observed in cluster 4 after the lesion. As in the control data, this cluster contained cells with negative spikes.

The relative depth of modulation of the spiking activity during the food retrieval task was compared between the control monkeys (L and G), and the lesioned monkey (P). Neural recordings in monkey O were gathered using a simplified task, which required only a modified lever to be pulled (see Materials and Methods); as this was not comparable to the full reach-and-grasp task, no measurements of task-related modulation in firing rate were made from this animal. There was no significant change in the relative depth of modulation for clusters 1, 2, and 4 in monkey P compared with control monkeys L and G; as noted above, this comparison could not be made for cluster 3 as only one cell was recorded in this cluster in the lesioned monkey.

After the lesioning, only nine cells had a significant peak in their DTT, and were assigned to cluster 2 (3 of 59 cells, 5%), cluster 3 (1 of 1 cell), and cluster 4 (5 of 20 cells, 25%). The overall proportion of significant peaks after lesioning was not significantly different from the control data (13.5% vs 7.2%, *p* > 0.05, χ^2^ test).

## Discussion

### Separation of activity into clusters

This report is the first to separate neurons in the primate reticular formation into different categories based on extracellular discharge. Previous attempts to subclassify extracellular recordings have typically used single measurements, in brain regions with clear separable cells. Examples of such an approach would be pyramidal neurons versus interneurons in rat cortex (Barthó et al., 2004). In the primate reticular formation, we did not find any single variable that could separate different cells. However, when six measures relating to the action potential and interspike interval were combined, there was evidence for separation into four clusters (Fig. 2).

It is important to accept that the use of four clusters is a provisional classification, based on the current dataset. The clustering was performed on a six-dimensional space, and as such it is impossible to gain an intuitive feel for how well this represents the data. However, projection onto the first two principal components (Fig. 2*O*) revealed clusters that do not have regions of empty space between them. Even measures that differed significantly between clusters were typically overlapping in their distributions, with area under the ROC curve varying from 0.64 to 1 (0.5 signifies perfect overlap). We have used different statistical tests to evaluate the number of clusters in our data. The kmeans and the linkage methods determined the optimal number of clusters in our data to be 2 and 4, respectively. The separation of our data points into four clusters was similar independent of the clustering method. This gave us confidence in using four clusters to segregate and provide a more complete characterization of our data. However, it is possible that different numbers of clusters would be assigned using a different dataset, or with different variables. We found that it was not possible to detect four clusters when using the more limited dataset from a single animal; our findings rely on using the larger number of cells only feasible by combining data across multiple animals. It is thus entirely possible that an even larger dataset would reveal evidence for a higher cluster number. This situation is probably no different from that in other brain areas: the simple dichotomy of pyramidal neurons versus interneurons in the cortex has progressively been replaced with an ever-increasing number of subclasses as understanding has advanced. For the primate reticular formation, we present a separation into four clusters as the first attempt at rational division of the neural population in this important motor center.

The different characteristics of the clusters may provide some information about their functional roles. Antidromically identified RST cells had narrow spikes, and the distribution of spike width was very similar to that of cluster 2 (Fig. 9*B*, purple vs blue). It is tempting therefore tentatively to assign cluster 2 to neurons that project to the spinal cord. Cluster 2 cells showed only small effects in spike-triggered averages of local field potential. This might be consistent with a role in transmitting information out of the reticular formation to spinal circuits, rather than in local circuit processing involving extensive synaptic interactions with neighboring cells (Denker et al., 2011; Einevoll et al., 2013). In primate motor cortex, the fast corticospinal neurons have narrow spikes, possibly due to the expression of K_v_3 channels, which are typically found only in fast-spiking interneurons in rodents (Vigneswaran et al., 2011; Soares et al., 2017). In monkeys, the RST has maximal conduction velocity similar to that in the corticospinal tract (Riddle et al., 2009). It is possible that narrow action potentials are an adaptation to fast conduction over long axon tracts, which would explain why we also find narrow spikes in antidromically identified RST neurons.

The RST is a diverse pathway, with reticulospinal axons using not just glutamate, but also glycine or GABA as a neurotransmitter (Du Beau et al., 2012). This permits monosynaptic inhibition of spinal neurons (Jankowska et al., 1968; Peterson et al., 1979). It is therefore perhaps surprising that identified reticulospinal cells should have overlapped so well with just one cluster in our population. It may be that reticulospinal cells are electrophysiologically homogeneous, with their intrinsic properties dictated primarily by the need to communicate rapidly with the spinal cord rather than by differences in neurotransmitter phenotype. Alternatively, it could be that we had a sampling bias. In the motor cortex, it is known that recordings from extracellular electrodes favor larger neurons with faster conduction velocity (Humphrey and Corrie, 1978). It is possible that inhibitory RST cells have a slower velocity than excitatory ones, leading to their under-representation in our sample here.

The shape of the spikes varied between the clusters. The first three clusters showed positive inflections (peaks). Cluster 4 contained cells with a negative maximal inflection (troughs) in the spike waveform. It is hard to speculate on the biophysical interpretation of the spike shapes, but we can, however, note that these cells in cluster 4 showed a clear separation from each of the other clusters in all the measures we tested (Fig. 3). Interestingly, clusters 1 and 2 also showed a significant difference in both spike width and normalized spike height (Fig. 3).

The clusters were also separable on the basis of their firing patterns. Cluster 1 cells were slowly firing. Cluster 3 had fast, regular spiking, reflecting a high incidence of peaks in their afterspike distance-to-threshold trajectory. The amplitude of these peaks was large compared with those that we previously reported in pyramidal tract cells in M1 (mean peak amplitude in reticular formation, 0.49 ± 0.07 vs 0.282 ± 0.015 in M1 pyramidal tract neurons (PTNs; Wetmore and Baker, 2004). Interestingly, cluster 3 cells tended to show less modulation with motor task than other clusters; this may be because the large peak in distance-to-threshold trajectory effectively clamped the firing at a fixed rate, preventing small fluctuations in response to changing synaptic input. Just as for M1, the presence of such cells with a strong propensity to rhythmic discharge could enhance local network oscillations. The median peak latency of 25 ms implies a preferred frequency of 40 Hz; our laboratory has recently gathered preliminary data suggesting that the reticular formation can exhibit oscillatory activity in this range (M. O. Cunningham, G. Collins, and S. N. Baker, personal communication).

### Changes in reticular formation cells after pyramidal tract lesion

One potential problem with searching for changes after recovery from a pyramidal tract lesion is that the cells were clustered using the same measures that were tested for changes. Clearly, if there had been large changes in any of the variables after lesioning, this could have shifted cells into a different cluster, rendering interpretation difficult. However, it seems unlikely that substantial numbers of cells were misclassified in the after-lesion data. The separation of clusters relied on six different measures. Changes in just one or two of these would not necessarily alter cluster assignments. For example, Figure 11, *G* and *H*, shows that cluster 1 (red) reduced its spike width substantially after the lesioning, from 2.3 to 1.8 ms. This shifted the spike width of the after-lesion cells for cluster 1 to be heavily overlapping with the other clusters. Yet, because other key features of the cluster 1 phenotype were unaltered, cells were still assigned to cluster 1. However, it is worth noting that cluster 3, which encompassed the highly rhythmic cells in the control data, significantly shrunk after the lesioning while the proportion of cells in cluster 4 doubled. These changes could reflect modifications in the nature of the cells compensating for the loss of the rhythmic cortical input.

Cluster 2 neurons, which may include reticulospinal cells, increased their firing rate after lesioning. The change in mode ISI was equivalent to a 60% increase in firing rate. Our previous work reported that reticulospinal synapses to motoneurons innervating intrinsic hand muscles increased the total size of EPSPs by 2.5-fold following recovery from a pyramidal tract lesion (Zaaimi et al., 2012). If this coincided with an increase of reticulospinal firing by 60%, the overall RST input to motoneurons would be almost four times greater. Our data, therefore, suggest that changes in the reticular formation operate alongside those within the spinal cord to restore lost input to motoneurons.

Some insight into the mechanisms underlying the rise in firing rate of cluster 2 cells may be provided by the two other significant changes that were observed after lesioning. Cluster 2 cells also showed a decrease in irregularity. This is likely to reflect changes in intrinsic conductances within the cell. Many PTNs provide collateral input to the reticular formation (Keizer and Kuypers, 1989). Given the level of our lesion, some of these would also have been interrupted, removing synaptic input from the reticular neurons. In the spinal cord, the loss of descending drive to motoneurons leads to enhanced persistent inward currents (PICs), effectively replacing excitatory synaptic input with depolarizing drive from PICs (Gorassini et al., 2004). A similar effect may have occurred here within the reticular formation after the loss of corticoreticular inputs. As PICs provide a more consistent drive compared with noisy synaptic input, in motoneurons this leads to a reduction in firing irregularity, as was seen here. Second, we also saw a rise in the firing rate of the cluster 1 cells. If our putative identification of these cells as local circuit interneurons is correct, it would imply that local circuits may also have been reconfigured to increase drive to the RST cells.
